# Development and Validation of a Microbiological Agar Assay for Determination of Orbifloxacin in Pharmaceutical Preparation

**DOI:** 10.3390/pharmaceutics3030572

**Published:** 2011-08-29

**Authors:** Edith C. L. Cazedey, Hérida R. N. Salgado

**Affiliations:** Programa de Pós-graduação em Ciências Farmacêuticas, Faculdade de Ciências Farmacêuticas, Univ Estadual Paulista, Rod. Araraquara-Jaú, km 1, CEP 14801-902,Araraquara, SP, Brazil

**Keywords:** orbifloxacin, fluoroquinolones, bioassay, validation

## Abstract

Orbifloxacin is a fluoroquinolone with broad-spectrum antimicrobial activity, and belongs to the third generation of quinolones. Regarding the quality control of medicines, a validated microbiological assay for determination of orbifloxacin in pharmaceutical formulations has not as yet been reported. For this purpose, this paper reports the development and validation of a simple, sensitive, accurate and reproducible agar diffusion method to quantify orbifloxacin in tablet formulations. The assay is based on the inhibitory effect of orbifloxacin upon the strain of *Staphylococcus aureus* ATCC 25923 used as test microorganism. The results were treated statistically by analysis of variance and were found to be linear (*r* = 0.9992) in the selected range of 16.0–64.0 μg/mL, precise with relative standard deviation (RSD) of repeatability intraday = 2.88%, intermediate precision RSD = 3.33%, and accurate (100.31%). The results demonstrated the validity of the proposed bioassay, which allows reliable orbifloxacin quantitation in pharmaceutical samples and therefore can be used as a useful alternative methodology for the routine quality control of this medicine.

## 1. Introduction

**Figure 1.**
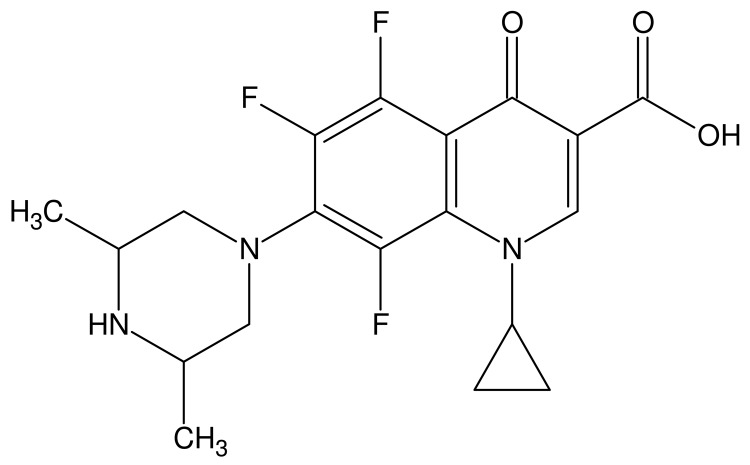
Chemical structure of orbifloxacin (CAS 113617-63-3).

The fluoroquinolones are a class of compounds that comprise a large and expanding group of synthetic antimicrobial agents. Orbifloxacin (Figure 1) (1-cyclopropyl-5,6,8-trifluoro-1,4-dihydro-7-(*cis*-3,5-dimethyl-1-piperazinyl)-4-oxoquinoline-3-carboxylic acid) is a new third-generation fluoroquinolone antibacterial drug, developed exclusively for the treatment of gastrointestinal and respiratory infections in animals [1-3], but it is also used for treatment of skin, soft tissue, and urinary tract infections (in dogs and cats, especially) [4,5]. In addition, research is still being carried out on the antimicrobial effects of this analyte. Recent studies have focused on the effectiveness of orbifloxacin in the treatment of staphylococcal pyoderma both superficial and deep [6], the antimicrobial activity of orbifloxacin against *Pseudomonas aeruginosa* isolated from canine otitis [7] and activity against *Staphylococcus intermedius* isolated from canine skin and ear infections [8]. Research is also being conducted on the possibility of widening the scope of orbifloxacin applications and using it for treatment of other animals, such as horses or rabbits [4,5,9].

Fluoroquinolones have become an important group, particularly for the treatment of infections caused by antibiotic-resistant bacteria [9].

Antimicrobial resistance of bacteria is a phenomenon that has been in constant evolution since the introduction of antibiotics. Several factors are known to promote bacterial resistance, including poor compliance with treatment regimen [10,11], prophylactic use of antibiotics [12] and the use of antibiotics as growth promoters [13,14], thus emphasizing the importance of dosage and appropriate treatment.

For an appropriate dosage of the pharmaceutical form orbifloxacin analytical methods should be developed and validated. To date, all methods described in the scientific literature for determination of this analyte in biological and others materials involve liquid chromatography, with UV detection [2,4,15-19], fluorimetry detection [5,19-27] or mass spectrometry [21-24,28] and a spectroscopic technique coupled to a flow system [29] as detection techniques.

There have been few reported methods for orbifloxacin, nor is there a method described in official compendia and no method for determination of orbifloxacin in dosage forms. Hence, an attempt has been made to develop a simple, sensitive, accurate and reproducible method for the determination of orbifloxacin in pharmaceutical dosage forms along with method validation.

## 2. Experimental Section

### 2.1. Chemicals

The orbifloxacin reference substance (assigned purity 99.8%) was supplied by Sigma-Aldrich. The pharmaceutical form tablets were commercially obtained and claimed to contain 22.7 mg orbifloxacin (Orbax^®^-Schering-Plough, Brazil). All reagents used were analytical grade. Purified water was used in all experiments.

### 2.2. Orbifloxacin Reference Solutions

An accurately weighed amount of powder equivalent to 5 mg of orbifloxacin reference standard was transferred to 50 mL volumetric flask and dissolved in 0.5 M hydrochloride acid solution to obtain final concentration of 100 μg/mL. Aliquots of this solution were diluted in the phosphate buffer solution pH 6.0 to give concentrations of 16.0, 32.0 and 64.0 μg/mL (S1, S2 and S3, respectively), which were used in the bioassay.

### 2.3. Preparation of the Sample Solutions

Twenty tablets were weighed and pulverized. An amount of powder equivalent to 10 mg of orbifloxacin was transferred to a 100 mL volumetric flask with 50 mL 0.5 M hydrochloride acid solution and shaken for 30 min in ultrasonic. This was followed by making up to volume with the same solvent. Aliquots of this solution were further diluted in phosphate buffer solution pH 6.0 to obtain the concentrations of 16.0, 32.0 and 64.0 μg/mL (T1, T2 and T3, respectively) which were tested against S1, S2 and S3.

### 2.4. Microorganism and Inoculum Standardization

The strain of *Staphylococcus aureus* ATCC 25923 shows to be the most appropriated test microorganism because of its susceptibility to orbifloxacin and capacity to form sharply defined inhibition of growth zones, allowing precision in the measurements.

The cultures of *S. aureus* ATCC 25923 were cultivated and maintained on Casoy culture medium (Difco, Brazil). The microorganism standardization was made according to the procedure described in the Brazilian and The United States Pharmacopeias [30,31]. Prior to use, the microorganism was grown in a BHI broth in a conical flask, which was incubated during 24 h at 35 ± 2 °C. Using a spectrophotometer with the wavelength set at 580 nm and a 10 mm absorption cell, the broth containing the microorganism was diluted to give a suspension with 25 ± 2% turbidity (transmittance) with the some broth sterile solution as the blank. From this standardized suspension, aliquots of 1.0 mL were added to each 100 mL of Grove–Randall's 11 culture medium (Difco, Brazil) at 48 °C, and used as the inoculated layer in the plate.

### 2.5. Agar Diffusion Bioassay

The base layer agar was composed of 20 mL Grove–Randall's 1 culture medium (Difco) that was poured into a 100 × 20 mm Petri dish [30,31]. After solidification of this layer, portions of 5 mL of inoculated Grove–Randall's 11 medium was poured onto the base layer.

**Figure 2.**
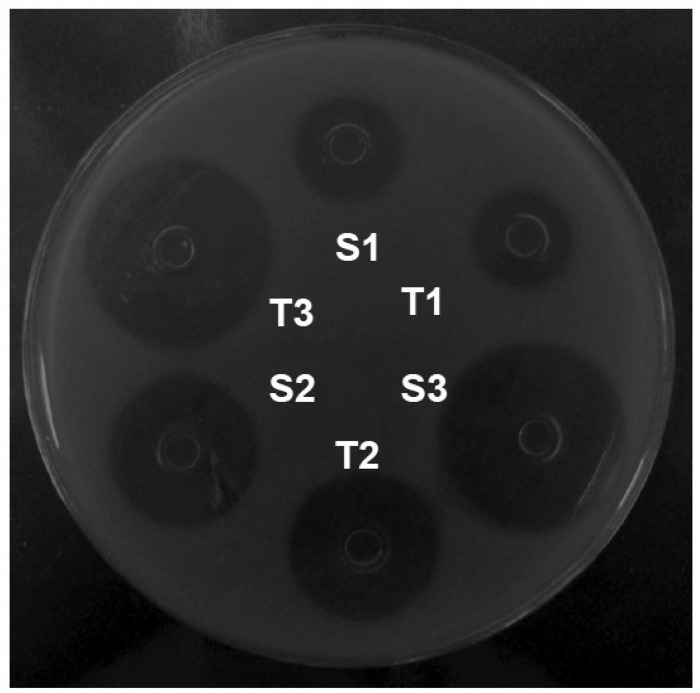
Agar diffusion assay (cylinder plate method) using a strain of *S. aureus* ATCC 25923 as the test microorganism. Orbifloxacin reference substance is at concentrations of 16 (*S1*), 32 (*S2*), and 64 (*S3*) μg/mL and orbifloxacin sample at concentrations of 16 (*T1*), 32 (*T2*), and 64 (*T3*) μg/mL.

In each plate, six stainless steel cylinders of uniform size (8 × 6 × 10 mm) were placed on the surface of the inoculated medium. Three alternated cylinders were filled with 200 μL of the reference solutions (S1, S2, and S3), and the other 3 cylinders were filled with the concentrations of the sample solutions (T1, T2, and T3; Figure 2). Six plates were used for each assay. The plates were incubated at 35 °C aerobically for 18 h. The growth inhibition zone diameters (mm) were carefully measured with a digital caliper.

### 2.6. Calculation of Activity and Method Validation

To calculate the activity of orbifloxacin, the Hewitt equation was used [32]. The assays were calculated statistically by the linear parallel model and regression analysis and verified using analysis of variance (ANOVA).

The method was validated by determination of the following operational characteristics: linearity, precision, accuracy, and robustness [31,33-39].

- *Linearity*—In order to assess the validity of the assay, 3 doses of the reference substance were used. The linearity was evaluated by linear regression analysis, which was calculated by the least-squares method.
- *Precision*—The precision of the method was determined by repeatability and intermediate precision and was expressed as the relative standard deviation (RSD). The repeatability was examined by assaying 7 samples of orbifloxacin on the same day (intraday) and under the same experimental conditions against the orbifloxacin reference standard. The intermediate precision of the method was evaluated through the performance of the analysis on 3 days (interday) and by having different analysts perform the analysis in the same laboratory (between-analysts).
- *Accuracy*—To determine the accuracy of the proposed method, the test was performed over 3 concentration levels, 80, 100 and 120%, covering the specified range. Accurately aliquots of 0.48, 0.8, and 1.12 mL of the reference standard solution (100 μg/mL) were transferred into 5 mL volumetric flasks together with aliquots of sample solutions (100 μg/mL) and diluted with phosphate buffer solution pH 6.0 to give final concentrations of 25.6, 32.0, and 38.4 μg/mL, respectively.
- *Robustness*—The robustness of the method was determined by analyzing the same sample under a variety of conditions. The factors considered were incubation time, volume of the inoculated layer (thickness), volume filled in cylinders, the solvent used for standard and sample dilution and the microorganism concentration on the inoculated layer.

## 3. Results and Discussion

The development and validation of analytical methods for the potency determination has received considerable attention in recent years, mainly from regulatory agencies, because of their importance in pharmaceutical analysis [36-39].

In this case, a microbiological assay was proposed as a suitable method for the determination of orbifloxacin in tablet forms.

The experimental conditions were tested and adjusted to accurately determine the performance of the assay. The strain of *Staphylococcus aureus* ATCC 25923 was found to be an appropriate test microorganism because of its sensitivity to orbifloxacin and its capacity to form sharply defined inhibition growth zones, allowing measurements with precision.

The potency of an antibiotic may be demonstrated under suitable conditions by comparing the growth inhibition of sensitive microorganisms induced by known concentrations of the antibiotic to be examined and a reference standard [30,31].

In this experimental work of 3 × 3 design (Figure 2), 3 dose levels for each standard and sample, were respectively used following the procedures described in the Brazilian Pharmacopoeia [30]. The calculation procedures normally assume a direct relationship between the observed zone diameter and the logarithm of the applied dose. The results of growth inhibition zone diameter of orbifloxacin reference substance are shown in Table 1.

The calibration curve for orbifloxacin was constructed by plotting zone diameter (mm) *versus* log of concentrations (μg/mL) and showed good linearity in the 16–64 μg/mL range.

The representative linear equation for orbifloxacin was *y* = 8.5263 Ln(*x)* + 9.2756 (*n* = 3, *r* = 0.9996), where *x* is log dose and *y* is zone diameter. The experimental values obtained for the determination of orbifloxacin in samples are presented in Table 1. According to the Brazilian [30] and The United States [31] Pharmacopeias, if a parallel-line model is chosen, the 2 log dose-response lines of the preparation to be examined and the reference preparation must be parallel, and they must be linear over the range of doses used in the calculation. These conditions must be verified by validity tests for a given probability, usually *P* = 0.05. The assays were validated by means of the ANOVA, as described in those official codes. There were no deviations from parallelism and linearity with the obtained results (*P* < 0.05).

The method precision in terms of repeatability (intra-assay) was evaluated by analyzing, on the same day, seven samples of orbifloxacin reference substance with same theoretical concentration. The intermediate precision was determined by analyzing the same sample on 3 days (between-day) with obtained RSD values of 2.88 and 3.33%, respectively. The orbifloxacin activity ranged from 99.18 to 101.84%, with a RSD value of 1.42%. The lower RSD values achieved confirm that the proposed method has capacity to generate, for the same sample, reproducible results with low response variation between independent assays.

The accuracy of the method was evaluated at 80, 100 and 120% of the nominal analytical concentration in the specified range of 16.0–64.0 μg/mL. The mean accuracy was 100.31% and RSD was 1.04% (Table 2), which confirms the ability of the method to determine with accuracy the orbifloxacin concentration within the range of 80–120% and, in the same way, shows that the results obtained from the bioassay were close to the true concentration values of the samples.

The quantification of antibiotic components by chemical methods, such as HPLC and UV spectrophotometry, although precise, cannot provide a true indication of biological activity. Attempts to correlate antibiotic bioassay results with those from chemical methods have proved disappointing. Therefore, bioassays continue to play an essential role in manufacturing and quality control of antibiotic medicines, and still demand considerable skill and expertise to assure success [40]. Although the biological assays can have a high variability, the analysis of the obtained results demonstrated that the proposed method might be very useful for determination of this drug in pharmaceutical dosage forms.

The results obtained in this study were very satisfactory, and the performed validation proved that microbiological assay is a good alternative methodology for pharmaceutical analysis of orbifloxacin in tablets. It is a useful analytical tool as a supplement or substitution for the physicochemical method.

## 4. Conclusions

The results indicated that the microbiological cylinder plate assay demonstrated good linearity, precision and accuracy at concentration ranging from 16.0 to 64.0 μg/mL, therefore, being an acceptable alternative method for the routine quality control of orbifloxacin in pharmaceutical forms. The method uses simple reagents, with minimum sample preparation procedures, and no toxic residues, encouraging its application in routine analysis.

## Figures and Tables

**Table 1.** Diameters of growth inhibition zones for orbifloxacin reference substance solutions obtained for standard curve.

**Concentration, μg/mL**	**Range of zone size, mm^a^**	**Mean diameters of growth inhibition zones,^b^ mm**	**RSD, %**
16.0	14.16–15.05	14.46	3.53
32.0	19.98–20.20	20.08	0.55
64.0	25.87–26.96	26.28	2.26

a Digital caliper resolution is 0.01 mm;

b Mean of 3 assays with 6 plates en each.

**Table 2.** Accuracy of the microbiological assay of orbifloxacin.

**Run**	**Amount of standard, μg/mL**		**Recovery,^a^%**	**RSD, %**

	**Added**	**Recovered**		
R1	0.480	0.487	101.50	
R2	0.800	0.798	99.76	1.04
R3	1.120	1.116	99.65	

a Mean of 3 assays.
